# Effect of a high value care curriculum on standardized patient exam in the Core Clerkship in Internal Medicine

**DOI:** 10.1186/s12909-020-02303-1

**Published:** 2020-10-15

**Authors:** Amit K. Pahwa, Kevin Eaton, Ariella Apfel, Amanda Bertram, Rebecca Ridell, Danelle Cayea

**Affiliations:** 1 Division of Hospital Medicine, Division of General Pediatrics, Johns Hopkins University School of Medicine, 600 North Wolfe Street, Baltimore, MD 21287 USA; 2 Divsion of General Internal Medicine, Department of Medicine, Johns Hopkins University School of Medicine, 600 North Wolfe Street, Harvey 806, Baltimore, MD 21287 USA; 3 Division of General Internal Medicine, Department of Medicine, Johns Hopkins University School of Medicine, 2024 East Monument Street, Baltimore, MD 21287 USA; 4 Division of General Internal Medicine, Department of Medicine, Johns Hopkins University School of Medicine, 601 North Caroline Street, Baltimore, MD 21287 USA; 5 Office of Assessment and Evaluation, Johns Hopkins University School of Medicine, 2024 East Monument Street, Room 1-200, Baltimore, MD 21287 USA; 6 Division of Geriatrics, Department of Medicine, Johns Hopkins University School of Medicine, 5200 Easter Avenue, Mason Lord Building Center Tower Suite, Baltimore, MD 2200 USA

**Keywords:** Clerkships, High value care, Medical student, Standardized patient

## Abstract

**Background:**

With almost 20% unnecessary spending on healthcare, there has been increasing interest in high value care defined as the best care for the patient, with the optimal result for the circumstances, delivered at the right price. The American Association of Medical Colleges recommend that medical students are proficient in concepts of cost-effective clinical practice by graduation, thus leading to curricula on high value care. However little is published on the effectiveness of these curricula on medical students’ ability to practice high value care.

**Methods:**

In addition to the standard curriculum, the intervention group received two classroom sessions and three virtual patients focused on the concepts of high value care. The primary outcome was number of tests and charges for tests on standardized patients.

**Results:**

136 students enrolled in the Core Clerkship in Internal Medicine and 70 completed the high value care curriculum. There were no significant differences in ordering of appropriate tests (3.1 vs. 3.2 tests/students, *p* = 0.55) and inappropriate tests (1.8 vs. 2.2, *p* = 0.13) between the intervention and control. Students in the intervention group had significantly lower median Medicare charges ($287.59 vs. $500.86, *p* = 0.04) and felt their education in high value care was appropriate (81% vs. 56%, *p* = 0.02).

**Conclusions:**

This is the first study to describe the impact of a high value care curriculum on medical students’ ordering practices. While number of inappropriate tests was not significantly different, students in the intervention group refrained from ordering expensive tests.

## Background

Compared to other developed countries, the United States has inferior health outcomes despite having the highest health care expenditure per capita in the world [1]. While some of the health expenditures could be attributed to higher prices, almost 20% is considered unnecessary spending [2, 3]. Because of this, there has been a movement to increase high value care (HVC), which is defined by the Institute of Medicine as the best care for the patient, with the optimal result for the circumstances, delivered at the right price [4].

Understanding the importance of HVC education in medical school, the American Association of Medical Colleges recommended that medical students demonstrate proficiency in the concepts of cost-effective clinical practice upon graduation [5]. As a result, most medical schools have required coursework on costs of care [6]. Yet, students and internal medicine clerkship directors feel education in these concepts is inadequate, and only 30% of internal medicine clerkships have a formal HVC curriculum [7, 8]. Additionally, most of the HVC curricula developed for clinical clerkships do not have an assessment [8–10].

This mismatch between assessments and curricula as well as the paucity of effective HVC curriculum still needs to be addressed. We aimed to study the effectiveness of a HVC curriculum in the Core Clerkship in Internal Medicine (IM Clerkship) using a randomized control trial and an outcome directly controlled by medical students.

## Methods

The study was conducted during the IM Clerkship at Johns Hopkins University School of Medicine (JHUSOM) from October 2014 – December 2015. The IM Clerkship at JHUSOM is a nine-week course that is offered five times in an academic year (blocks 1, 2, 3, 4, 5) to third year medical students. The study began in block 2 of academic year 2015 (AY15). For purposes of randomization, blocks were grouped into two groups of three (Group 1: B2-AY15, B4-AY15, B1-AY16, Group 2: B3-AY15, B5-AY15, B2-AY15). Group 2 was randomly selected to be the intervention group. All students who were enrolled in the IM Clerkship during the intervention quarters received the curriculum. Enrollment was done through JHUSOM registrar’s office, which was not involved in the study. All students in the intervention group participated in the HVC curriculum. Prior to beginning the IM Clerkship, regardless if it was a control or intervention group, a research coordinator described the study and students signed an informed consent opting in or out of data analysis. Students were not explicitly told if they were receiving the HVC curriculum or not. The control and intervention groups were compared using several types of demographic data. Comparisons of gender, ethnicity, race, and undergraduate major between control and intervention groups were made using chi-square or Fisher’s exact tests, as appropriate. Comparisons of undergraduate grade point average (GPA) and age between control and intervention groups were made using the Mann-Whitney *U* test. Comparisons of Medical College Admission Test® (MCAT®) scores were made using an independent samples *t* test. All tests were two-sided, and *p*-values < 0.05 were considered statistically significant. Analyses were conducted using SAS Enterprise Guide 6.1 (SAS Institute Inc.; Cary, NC). To maintain confidentiality of students, only the research coordinator had access to individual data. Once grades were finalized for the clerkship, the study team was able to see the data only in aggregate form.

The HVC curriculum consisted of two classroom sessions and online modules. One classroom session focused on cost-effective analysis (CEA). Students were first introduced to the concept and basic terms of CEA and then participated in a small group discussion on a CEA of noninvasive cardiac stress testing. The second classroom session focused on hospital charges. In small groups, students wrote hospitalization orders for a patient with history of congestive heart failure presenting with shortness of breath. Once completed, they were given the Johns Hopkins Hospital (JHH) charges for each of their orders in order to compare how much one group spent compare to the other group. Each of the classroom sessions had a post-session evaluation that contained common questions for all sessions in the clerkship as well as questions regarding specific individual sessions.

Lastly, students were provided with online modules that consisted of managing three patients with common symptoms derived from the Clerkship Directors of Internal Medicine (CDIM) – Society of General Internal Medicine (SGIM) Core Medicine Clerkship Curriculum Guide 3.0 [11]. For each of the virtual patients, students had a fixed budget to care for them. After placing an order, the program deducted the 2014 Medicare allowable fee from the budget. Upon completion of the modules, the program displayed the appropriateness and charges of the students’ management plans. Four internal medicine faculty members and one subspecialist (in the area of the patient’s symptoms) a priori through the Delphi method determined the appropriateness of the answer choices available to students. None of these faculty were involved in development of the HVC curriculum.

During the clerkship, students could rotate at three different sites but all students were assigned to JHH at some point during the clerkship. While at JHH, they have an assigned teaching attending who conducts sessions with them at various times during the rotation. Faculty during the intervention blocks were given specifics about the curriculum and encouraged to incorporate HVC into their sessions with the students. At the end of each rotation students completed a survey through the SurveyMonkey® platform assessing their perception of education in HVC as well as their experiences on the clerkship. This survey was used as part of a needs assessment in a previous study [7].

The primary outcome of curricular efficacy was student performance on a standardized patient exam (SPE) at the end the clerkship, including appropriate and inappropriate orders as well as the Medicare allowable fees for each test. The SPE is part of the summative assessment for the clerkship that was created prior to the study. All students in both the intervention and control group completed the same cases in the SPE. The cases in the SPE did not have similar presenting symptoms or diagnoses to the classroom-based sessions or the online modules. Student’s management choices were assessed on appropriateness for the clinical presentation. Secondary outcome was performance on the National Board of Examiners (NBME) subject exam.

Statistical analysis of assessment and survey data was performed with SAS 9.4. Two sample t-tests were run comparing the number of inappropriate tests as well as the number of appropriate tests between control and intervention groups. Additionally, a Kruskal-Wallis test was run to compare the total cost of these tests (which was not normally distributed) between the intervention and control group and a Wilcoxon Rank-Sum test was run to compare the total cost of these tests and NBME percentile scores (which was not normally distributed) between the intervention and control group.

## Results

During the study period, 136 students completed the IM Clerkship. Of those 136 students, 70 (intervention) received the HVC curriculum and 66 (control) received the standard education. There were no significant differences between the two groups with respect to gender, ethnicity, race, undergraduate major, undergraduate GPA, or MCAT® score (Table 1). Students in the intervention group were older compared to the usual education group (26 vs 25 years old, *p* = 0.007). Including classroom time, the total HVC curriculum time was less than 4 h. No student opted out of having their assessment data used in the study.

**Table 1:** Student characteristics by control and intervention groups

Characteristic^a^	Control (***n*** = 66)	Intervention (***n*** = 70)	***p***-value
Gender			
Female	33 (50.0)	38 (54.3)	0.62
Male	33 (50.0)	32 (45.7)	
Ethnicity			
Hispanic	7 (10.6)	5 (7.1)	0.48
Non-Hispanic	59 (89.4)	65 (92.9)	
Race^b^			
American Indian or Alaska Native	1 (1.5)	1 (1.4)	1.00
Asian	23 (34.9)	23 (32.9)	0.81
Black or African American	11 (16.7)	4 (5.7)	0.06
Native Hawaiian or Other Pacific Islander	0 (0.0)	0 (0.0)	–
White	30 (45.5)	37 (52.9)	0.39
Undergraduate Major^c^			
Non-Science	14 (21.5)	16 (22.9)	0.85
Science	51 (78.5)	54 (77.1)	
Undergraduate GPA^d^			
Biology, Chemistry, Physics, and Math Courses	3.87 (3.76–3.93)	3.92 (3.81–3.98)	0.06
Total	3.88 (3.79–3.94)	3.91 (3.81–3.96)	0.07
MCAT Scores^e^	35.1 (32.1–38.1)	35.7 (32.5–38.8)	0.28
Age^f^ (years)	25.0 (24.0–26.0)	26.0 (24.0–28.0)	0.007

*GPA* Grade point average, *MCAT* Medical College Admission Test

^a^ Values for gender, ethnicity, race, and undergraduate degree are presented as *n* (%). Values for undergraduate grade point average and age are presented as median (inter-quartile range). Values for Medical College Admission Test scores are presented as mean (95% confidence interval)

^b^ Students were permitted to select all that apply, such that the sum of within-cell sample sizes may exceed the total group sample size

^c^ Undergraduate major was categorized as either non-science or science. Major information was unavailable for one student in the control group

^d^Ungraduate grade point average from the students’ American Medical College Application Service (AMCAS) application

^e^All students in this sample completed the test prior to the current version released in 2015. The scores presented represent the sum of the biological science, physical science, and verbal reasoning domain scores

^f^Age was defined as the students’ age at start of the clerkship

There were no significant differences in ordering of appropriate tests (3.1 vs. 3.2 tests/students, *p* = 0.55) and inappropriate tests (1.8 vs. 2.2, *p* = 0.13) between the intervention and control group. Students in the intervention group had significantly lower Medicare allowable fees ($287.59 vs. $500.86, *p* = 0.04). For secondary outcomes, students in the intervention achieved higher percentiles on the NBME subject exam (78% vs. 67%, *p* = 0.10), but this was not statistically significant (Table 2).

**Table 2:** Student performance on end of clerkship assessments

Assessment		Control (66)	Intervention (70)	p
Standardized patient	Appropriate tests/student (SD)	3.2 (0.94)	3.1 (0.90)	0.55
	Inappropriate tests/student (SD)	2.2 (1.5)	1.8 (1.3)	0.13
	Median Medicare allowable fees/student (IQR)	$500.86 ($227.90 – $870.33)	$287.59 ($169.87–681.18)	0.04
Median NBME percentile (IQR)		67 (42–88)	78 (59–92)	0.10

*SD* Standard Deviation

*IQR* Interquartile Range

*NBME* National Board of Medical Examiners

Response rate for the post-rotation survey was 60% (42/70) for intervention and 56% (37/66) for control. Overall, more students in the intervention group felt their education in HVC was appropriate (81% vs. 56%, *p* = 0.02). There were no significant differences in how often students perceived unnecessary ordering of tests, their comfort level speaking up about an unnecessary test, or how often they witnessed discussion of costs, praise for not ordering an unnecessary test, or guidance to order tests that will not change management (Table 3).

**Table 3:** Student perceptions of high value and low value care during the clerkship

Question	Answer	Control 37 (56%)	Intervention 42 (60%)	p
HVC Education	Appropriate	21 (57%)	34 (81%)	0.02
	Inadequate	16 (43%)	8 (19%)	
How Often Unnecessary Tests Ordered	Frequent/Often	10 (27%)	15 (36%)	0.41
	Sometimes/Rarely/Never	27 (73%)	27 (64%)	
Comfort Level Speaking Up Against Ordering Perceived Unnecessary Tests	Comfortable	16 (43%)	17 (41%)	0.80
	Uncomfortable	21 (57%)	25 (60%)	
How Often Discuss Costs	Frequent/Often	6 (17%)	9 (21%)	0.59
	Sometimes/Rarely/Never	30 (83%)	33 (79%)	
How Often Praised for Not Ordering an Unnecessary Test	Frequent/Often	16 (44%)	15 (36%)	0.43
	Sometimes/Rarely/Never	20 (56%)	27 (64%)	
How Often Team was Guided to Order More (unnecessary) Tests	Frequent/Often	8 (22%)	3 (7%)	0.07
	Sometimes/Rarely/Never	29 (78%)	38 (93%)	

*HVC* High Value Care

Most students rated the classroom sessions very good or excellent (75% for CEA and 90% for hospital charges session). Most students felt that the classroom sessions increased skills related to the topic somewhat or a lot (90% for CEA and 93% for hospital charges session) and prepared them to be a medical student on the IM clerkship. After the hospital charges sessions, most students would consider charges somewhat or a lot for laboratory tests (90%), medications (91%), and radiologic procedures (93%).

For the web-based modules, 88% of students rated them as good or excellent. For each individual module, students rated the following modules as good or excellent: acute kidney injury (85%), acute chronic obstructive pulmonary disease exacerbation (83%), acute deep vein thrombosis and pulmonary embolus (85%). Most students felt the virtual patients were similar to patients that they cared for during the clerkship yet only half (50%) felt virtual patients should be a part of the clerkship.

## Discussion

This is the first study to describe the impact of a HVC curriculum on medical students’ ordering practices as opposed to monitoring others’ care practices [12]. Students were highly satisfied with the curriculum and reported improved confidence in the adequacy of their HVC education. Students agreed that the virtual patients and topics had fidelity and were similar to what they see on the clerkship. The curriculum is also aligned with the CDIM-SGIM Core Medicine Clerkship Curriculum. Given the limited curriculum time and resources, implementation of this HVC curriculum at other schools is feasible. The use of virtual patients and small group learning as the main educational methods promotes active learning and limits the need for faculty time, which was cited as a barrier to HVC curriculum implementation by clerkship directors [8].

Students in high intensity regions are more likely to display low-value behaviors thus high value care should be taught as early as possible [13]. The healthcare intensity is a measure developed by the Dartmouth Atlas the amount of care a Medicare beneficiary received in the last 2 years of their life. For JHH, the healthcare intensity is approximately the 50th percentile [14]. While not the highest intensity, we feel the modeling may have led the students in the control group to spend more on testing. This pattern is seen in residents who train in higher intensity regions are more likely to spend more even if they move to a low intensity region [15].

This study demonstrated that medical students can apply concepts taught to them in simulated clinical scenarios which are not part of the formal curriculum. Because of this finding we have broadened our curriculum to include an intersession for first year medical students in an effort to inculcate students in HVC principles at an earlier stage [16].

The study did have some limitations. As with many educational interventions it was a single site study in the United States (US) thus we cannot be entirely sure if this curriculum would succeed at other medical schools in the US and outside the US. The assessment was not in the context of actual patient care but consisted of an exam with a simulated patients. As students often report standardized patient encounters feel somewhat unrealistic, this may have lead them to behave differently than they would do with real patients [17]. Therefore it is unknown how students would incorporate their knowledge on HVC in actual patient care. Lastly the effects were only assessed immediately after the IM clerkship so we are unable to determine if students will continue to less expensive testing beyond the third year of medical school.

## Conclusions

A curriculum in HVC can increase the perception of appropriateness of HVC education, and teach students to spend less money on patient care. While there is a need for curriculum in HVC in medical school, assessment of formal curriculum changes should always be performed. Having a repository of curricula and assessment tools for HVC will help medical schools in this regard [18–20]. Future studies will need to better delineate methods for measuring impact of HVC curricula at multiple institutions as well as beyond medical school.

## Data Availability

The datasets used and analyzed during the current study are available from the corresponding author on reasonable request.

## Abbreviations

CDIM: Clerkship Directors of Internal Medicine
CEA: Cost-effectiveness analysis
GPA: Grade point average
HVC: High value care
IM Clerkship: Core Clerkship in Internal Medicine
JHUSOM: Johns Hopkins University School of Medicine
JHH: Johns Hopkins Hospital
MCAT®: Medical College Admission Test®
NBME: National Board of Medical Examiners
SGIM: Society of General Internal Medicine
SPE: Standardized patient exam
US: United States
